# *PpSAUR5* promotes plant growth by regulating lignin and hormone pathways

**DOI:** 10.3389/fpls.2024.1291693

**Published:** 2024-06-25

**Authors:** Xin-Miao Li, Han-Han Zhai, Xiu-Hong An, He Zhang, Xueying Zhang, Pengfei Wang, Haijiang Chen, Yi Tian

**Affiliations:** ^1^ College of Horticulture, Hebei Agricultural University, Baoding, Hebei, China; ^2^ National Engineering Research Center for Agriculture in Northern Moutainous Areas, Agricultural Technology Innovation Center in Mountainous Areas of Hebei Province, Hebei Agricultural University, Baoding, Hebei, China

**Keywords:** *SAUR*, peach, phytohormone, tree vigor, transcriptome

## Abstract

**Introduction:**

Peach (*Prunus persica*) has a high nutritional and economic value. However, its overgrowth can lead to yield loss. Regulating the growth of peach trees is challenging. The small auxin-up RNA (*SAUR*) gene family is the largest family of auxin-responsive genes, which play important roles in plant growth and development. However, members of this gene family are rarely reported in peach.

**Methods:**

In this study, we measured leaf area, chlorophyll and lignin content to detect the role of *PpSAUR5* on growth through transgenic *Arabidopsis*.

**Results:**

*PpSAUR5* responds to auxin and gibberellin, promoting and inhibiting the synthesis of gibberellin and auxin, respectively. The heterologous transformation of *PpSAUR5* in *Arabidopsis* led to enhanced growth of leaves and siliques, lightening of leaf color, decrease in chlorophyll content, increase in lignin content, abnormalities in the floral organs, and distortion of the inflorescence axis. Transcriptome data analysis of *PpSAUR5* overexpression and wild-type lines revealed 854 differentially expressed genes (DEGs). GO and KEGG analyses showed that the DEGs were primarily involved in biological processes, such as cellular processes, metabolic processes, response to stimuli, and catalytic activity. These genes were mainly enriched in pathways, such as phenylalanine biosynthesis, phytohormone signaling, and MAPK signaling.

**Discussion:**

In summary, these results suggested that *PpSAUR5* might regulate tree vigor by modulating the synthesis of auxin and gibberellin. Future studies can use *PpSAUR5* as a candidate gene to elucidate the potential regulatory mechanisms underlying peach tree vigor.

## 1 Introduction

Peach (*Prunus persica*) is an important economic fruit tree with a wide cultivation area. However, the new shoots of peach trees grow vigorously. Improper management of peach trees might lead to excessive growth, leading to a burden on tree nutrition and, consequently, a decrease in yield. In orchard production, the vigor of peach tees is regulated by pruning or spraying the trees with growth regulators, which balances the vegetative growth and reproductive growth while increasing the production cost (Li et al., 2020). Therefore, it warrants the need to find novel, more cost-effective ways to efficiently manage peach tree growth. To date, a limited number of studies have focused on the regulatory mechanisms of peach vigor-related genes.

Phytohormones play crucial roles in regulating plant growth and development. Among them, auxin and gibberellin are essential for several developmental processes in plants, such as organ elongation (Tao et al., 2008; Sandalio et al., 2016); apical hook curvature growth and root hair development (Lee and Cho, 2013; Béziat and Kleine-Vehn, 2018); response to temperature (Gray et al., 1998) and light signals (Stepanova et al., 2008); and seed germination, flowering induction, and pollen maturation (Achard and Genschik, 2009). Moreover, these hormones interact with each other too. Ross et al. (2000) showed that exogenous auxin treatment reduces bioactive gibberellin (GA_1_) contents in pea stems. In *Arabidopsis*, fertilization-induced auxin activates GA biosynthesis; however, the application of exogenous GA does not affect auxin synthesis (Dorcey et al., 2009). SlDELLA, a GA signaling inhibitor, interacts with auxin signaling components SlIAA9 and SlARF7 to regulate tomato fruit development (Hu et al., 2018).

The *SAUR* family is an auxin-responsive, plant-specific, and extremely abundant gene family (Stortenbeker and Bemer, 2019). *SAUR* genes were originally identified in soybean hypocotyls and are extremely sensitive to exogenous auxin (McClure and Guilfoyle, 1987). Members of this gene family have been identified in several plants, such as *Arabidopsis*, tobacco, and maize (Newman et al., 1993; Gil et al., 1994; Knauss et al., 2003). Notably, *SAUR* genes have been found to exhibit similar functions across different plant species and primarily mediate cell elongation. For instance, *SAUR53* promotes cell and organ elongation in *Arabidopsis* and modulates apical hook development; however, it is not induced by exogenous auxin (Kathare et al., 2018). Overexpression of the *SAUR19–24* genes enhances cell expansion, increases hypocotyl length and leaf size, and alters overall plant growth (Xie et al., 2015). In maize and soybean, *SAUR* family genes are primarily expressed in elongated tissues, suggesting that these genes are involved in cell elongation. Another study reported that *SAUR36* is significantly less expressed in young leaves than in old leaves, and *saur36* mutant *Arabidopsis* plants exhibit a significant delay in leaf senescence, indicating the involvement of this gene in promoting leaf senescence (Hou et al., 2013). In a more recent study, treatment of *SAUR10*-clade transgenic *Arabidopsis* with different hormones showed that *SAURs* respond to auxin (indole-3-acetic acid, IAA), gibberellic acid (GA), abscisic acid (ABA), ethylene, and cytokinin (van Mourik et al., 2017). In rice, *OsSAUR39* and *OsSAUR45* have been shown to modulate plant growth by inhibiting auxin synthesis and transport (Kant et al., 2009; Xu et al., 2017). In addition, *SAUR* genes also respond to signals such as drought, low temperature, pests, and diseases.

Auxin activates plasma membrane (PM) H^+^-ATPase, mediating proton outflow. The hydrogen bonds in cell walls break under acidic conditions. In addition, an acidic environment changes the activity of cell wall-modified protein, leading to the degradation of the bond between xylan and cellulose microfibrils and cell wall relaxation (Xia et al., 2019). *SAUR* genes promote cell expansion by activating PM H^+^-ATPase via an acidic growth mechanism. Previous studies have shown that there is an antagonistic relationship between SAUR proteins and D-type protein phosphatase 2C (PP2C.D), a plant growth-related protein. For instance, the auxin-induced SAUR19 protein inhibits PP2C.D (D-type protein phosphatase 2C) activity (Spartz et al., 2017). *pp2c.d2* and *pp2c.d5* derivatives have been shown to constitutively dephosphorylate and inhibit PM H^+^-ATPases, restricting plant cell elongation and organ growth (Wong et al., 2019). Our previous studies showed that *PpSAUR5* expression is positively correlated with peach tree vigor. Therefore, *PpSAUR5* could be considered as a candidate gene for regulating tree vigor in peach. In the present study, we constructed *PpSAUR5*-overexpressing *Arabidopsis* plants using the *Agrobacterium*-mediated method. Phenotype, organ length, and chlorophyll and lignin contents were determined in transgenic lines. Furthermore, the responses of the transgenic lines to different hormone treatments were studied, and transcriptome analysis was performed on wild-type and *SAUR5–8* overexpression lines to determine the relationship between *PpSAUR5* expression and the growth potential of peach trees.

## 2 Materials and methods

### 2.1 Plant materials and growth conditions

*Arabidopsis thaliana* Col-0 was used for the transgenic assay. *Arabidopsis* seeds were disinfected and sown in Murashige and Skoog (MS) medium. When the seedlings had developed three to four leaves, the seedlings were transplanted to soil and placed in a climate incubator with a 16-h/8-h photoperiod at 23°C.

*Nicotiana benthamiana* was used for luciferase complementary assay. Seeds were sown in soil and placed in a climatic incubator with a 16-h/8-h photoperiod, humidity of 80% at 23°C.

### 2.2 Gene cloning and vector construction

Total RNA was extracted from the shoots of peach cultivar ‘Jiuyan’ using the RNAprep Pure Plant Kit (Tiangen Biotechnology, Beijing, China). Then, cDNA was synthesized using the TRUEscript RT Master Mix Kit (Adelaide Biotechnology, Beijing, China). Polymerase chain reaction (PCR) was performed using the cDNA as the template, and the reaction protocol was as follows: 98°C for 2 min; 33 cycles of 98°C for 10 s, 60°C for 15 s, 68°C for 30 s; 68°C for 10 min; and stored at 4°C. The PCR products were detected using 1% agarose gel electrophoresis, and the target bands were recovered using the Takara Gel Recovery Kit (TaKaRa Biotechnology, Dalian, China). The obtained DNA target fragment was ligated to the pEASY^®^-Blunt Cloning Vector and sequenced to validate its accuracy. Then, the DNA target fragment was inserted into the expression vector pRI-101. The primers used are shown in **Supplementary Table S1**.

### 2.3 Quantitative real time-PCR

Quantitative real time-PCR (qRT-PCR) was performed using 2x AugeGreen qPCR Master Mix (US EVE-RBRIGHT® INC). The reaction mix included 10 μL of 2x Fast Super EvaGreen® Master Mix, 2 μL each of forward and reverse primers (10 μM each), 2 μL of cDNA, and 4 μL of ddH_2_O. The qRT-PCR protocol was as follows: 95°C for 60 s, followed by 45 cycles of 95°C for 10 s, 58°C for 10 s, and 72°C for 10 s. The dissolution curve program was as follows: 95°C for 10 s, 65°C for 60 s, and 97°C 10 s. Relative gene expression was calculated using the 2^−ΔΔCT^ method. There were 10 genes randomly selected for qRT-PCR to verify the accuracy of transcriptome data. The internal reference gene was *ubq5*. The primers used are shown in **Supplementary Table S2**.

### 2.4 Yeast two-hybrid assay and luciferase complementary assay

The yeast two-hybrid (Y2H) expression vectors pBT3-STE and pPR3-N were used for the Y2H assay. The bait and prey plasmids were co-transformed into the yeast strain *NMY51*. Then, the strains were spread on SD/-Trp-Leu-deficient medium (DDO) and incubated at 28°C for 2–3 days. The clones were then spread onto SD/-Trp-Leu-Ade-His (QDO) and SD/-Trp-Leu-Ade-His + X-α-gal (QDO/X/A) media and incubated for 3–4 days to validate the interaction.

The expression vectors pCAMBIA1300-NLuc (pNL) and pCAMBIA1300-CLuc (pCL) were used for the luciferase complementation assay. From these vectors, recombinant plasmids pCAMBIA1300-PP2C.D2-NLuc and pCAMBIA1300-SAUR5-Cluc were constructed. The empty vector and the recombinant plasmid were transformed into *Agrobacterium GV3101*. PP2C.D2-Nluc + SAUR5-Cluc was the experimental group. PP2C.D2-Nluc + pCL, SAUR5-Cluc + pNL, and pNL + pCL were used as the control groups. Four-week-old vigorously growing *Nicotiana benthamiana* was selected as the material. 10 mM 2-morpholinoethanesulfonic acid (MES), 10 mM MgCl_2_·6H_2_O, and 10 mM acetosyringone (AS) were mixed to prepare the resuspension. The OD value of resuspended bacteria was 0.6. The four groups of mixed bacterial solutions were injected into the abaxial surface of the *Nicotiana benthamiana* leaves in different areas, and the leaves were observed after 2–3 days of incubation.

### 2.5 Transgenic *Arabidopsis* assay

The expression vector *pRI-PpSAUR5* was transformed into *Agrobacterium GV3101* using the floral dip method (Clough and Bent, 1998). The seeds of T0 generation were sown in MS medium (supplemented with 50 mg/L kanamycin). After two resistance screenings, if all the seeds germinated and survived, they were considered pure overexpression lines. For subsequent analyses, T2 generation plant DNA was used as the experimental template, expression plasmid DNA was used as the positive control, and wild-type *Arabidopsis thaliana* DNA was used as the negative control. The upstream and downstream primers were designed for PCR detection. The upstream primer was GCTCCTACAAATGCCATCA, and the downstream primer was TTACCAAATTGTTTTATCTGAAAAT.

### 2.6 Assessment of phenotypic characteristics

*Arabidopsis* seeds were screened for two generations to obtain homozygous overexpression lines. Three overexpression lines, *SAUR5–8*, *SAUR5–9*, and *SAUR5–15*, with varying *PpSAUR5* expression levels, were selected for further analyses. The hypocotyl length, root length, total chlorophyll content and levels of auxin and gibberellin in the lines were determined at 7 days after germination. The rosette leaf disc diameter and leaf area were measured at 20 days post-germination. Furthermore, we measured the length and number of stigmas and stamens at 45 days using an anatomical microscope (Zeiss Stereo Microscope Stemi 2000).

### 2.7 Analysis of *PpSAUR5* response to phytohormones

Media with varying concentrations of exogenous hormones were prepared. Separate media were prepared containing 50 nM, 75 nM, and 100 nM IAA; 50 μM, 100 μM, and 150 μM GA; and 10 μM and 20 μM N-1-naphthylphthalamic acid (NPA). Wild-type and transgenic *Arabidopsis* seeds were sown in MS medium, and seeds with consistent germination were picked and placed vertically in hormone-supplemented media. The root length of 7-day *Arabidopsis thaliana* was measured. Root length inhibition rate (%) = [(control group-experimental group)/control group].

### 2.8 Determination of hormone levels

Endogenous hormone levels were determined in wild-type and transgenic *Arabidopsis* lines using high-performance liquid chromatography. Briefly, 1 g of plant sample (*Arabidopsis* at 7 days) was weighed and added to a mixture containing 8 mL of 80% methanol solution and the antioxidant butylated hydroxytoluene (BHT). The sample was ground, homogenized, and extracted for 12 h at 4°C in the dark. The mixture was then subjected to ultrasonic treatment for 1 h on ice and centrifuged at 12,000 rpm for 15 min at 4°C. The supernatant was transferred to a fresh centrifuge tube, and nitrogen was blown into the aqueous phase. The pH of the aqueous phase was adjusted to 8.0 with 1 mol/L Na_2_HPO_4_ solution. Then, the sample was mixed with 0.2 g polyvinylpyrrolidone (PVPP), sonicated on ice for 0.5 h, and centrifuged at 12,000 rpm at 4°C for 5 min. The supernatant was transferred to a fresh centrifuge tube. The pH of the supernatant was adjusted to 3.0 with 1 mol/L citric acid. The extract was obtained with 3 mL ethyl acetate, ultrasonically shaken for 10 min, and then incubated in the dark for 1.5 h–2 h. The upper liquid layer was transferred to a fresh centrifuge tube and blown with nitrogen. When ethyl acetate was blown dry, 2 mL of 20% methanol was added to the residue for redissolution. An activated C18 column was rinsed with methanol and then with ultrapure water. The redissolved sample was transferred to a C18 column followed by 10% methanol rinse. Then, the column was again rinsed with 1 mL methanol at pH 8.0 and the liquid was collected into the injection bottle.

### 2.9 Determination of chlorophyll content

The 7-day *Arabidopsis* was ground into powder in liquid nitrogen and added to a mixture of acetone and anhydrous ethanol (2:1). After incubating at 4°C for 12 h, the solution was centrifuged at 4,000 rpm for 5 min at room temperature, and the absorbance values of the supernatant were measured at 645 nm and 663 nm.

### 2.10 Determination of lignin content

The lignin content in the plants was measured as described previously, with some modifications (Van Acker et al., 2013). The 20-day *Arabidopsis* was dried at 80°C to a constant weight, crushed, and passed through a 50-mesh sieve. Next, 5 mg of this sample was transferred to a 1.5-mL centrifuge tube. Then, 500 μL of 25% bromohexanoyl-acetic acid and 20 μL of 70% perchloric acid were added to the tube. The tube was then sealed with a sealing film, and the contents were fully mixed. The solution was then incubated in a water bath at 80°C for 40 min for sample acetylation. During the incubation, the sample was shaken every 10 min. After incubation in the water bath, the sample cooled naturally. Then, 500 μL of 2 M sodium hydroxide was added to the tube and mixed thoroughly. The tube was then left to stand. Finally, the absorbance value (A) of the sample was measured at 280 nm. The lignin content was measured using the following formula:


$$\text{Percentage of lignin }\left(\%\right)=[(A_{assay}–A_{blank})\times 0.2184]/W$$

Here, A_assay_ and A_blank_ are the absorbance values of the assay and the blank, respectively, and W is the sample mass.

## 3 Results

### 3.1 Effects of *PpSAUR5* overexpression on plant growth

**Figure 1:**
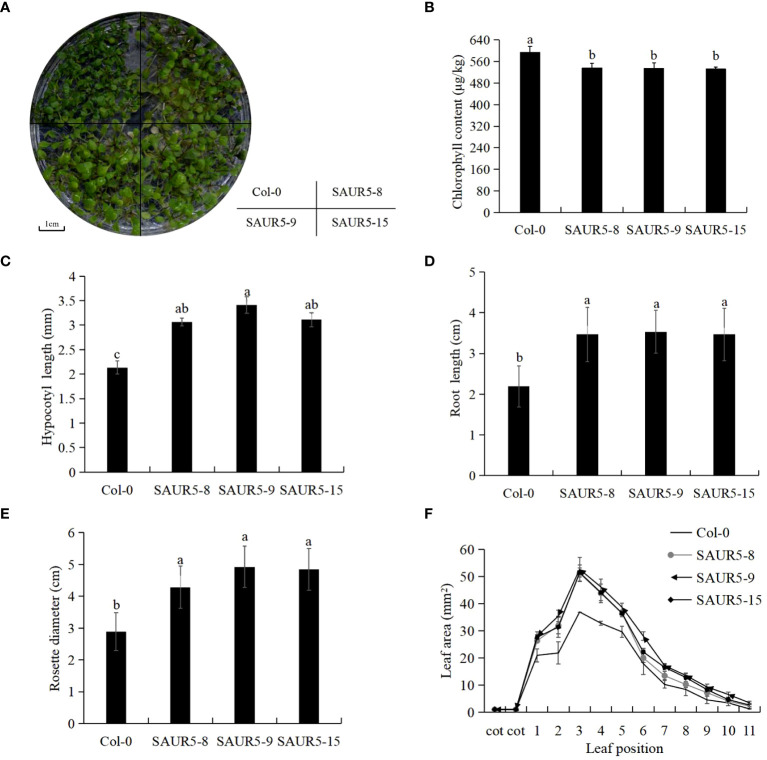
Vegetative growth phenotype of Col-0 and overexpressed *Arabidopsis*. Each data point represents mean of the data set and bars represent standard deviation (n = 20). Letters indicate significant differences by t test (P = 0.05). **(A)** 7-day growth of Col-0 and *PpSAUR5* overexpressed *Arabidopsis*. **(B)** 7-day Col-0 and *PpSAUR5* overexpress *Arabidopsis* chlorophyll content. **(C)** 7-day Col-0 and *PpSAUR5* overexpression *Arabidopsis* hypocotyl length. **(D)** 7-day Col-0 and *PpSAUR5* overexpression *Arabidopsis* root length. **(E)** 20-day Col-0 and *PpSAUR5* overexpressed *Arabidopsis thaliana* rosette leaves diameter. **(F)** 20-day Col-0 and *PpSAUR5* overexpress *Arabidopsis* leaf area.

The overexpression lines exhibited markedly enhanced plant growth, longer petioles, and lighter leaf color than Col-0 (**Figure 1A**, **Supplementary Figure S1**). Furthermore, the overexpression lines exhibited lower total chlorophyll content and significantly elongated hypocotyls and roots than Col-0 (**Figures 1B–D**). In addition, the transgenic lines exhibited significantly enhanced rosette leaf disc diameter and leaf area than wild-type plants. The largest leaf area measured in Col-0 was 36.98 mm^2^, whereas that measured in the overexpression lines was >50 mm^2^ (**Figures 1E, F**). These findings indicated that *PpSAUR5* overexpression modulated the vegetative growth of *Arabidopsis*.

### 3.2 Effects of *PpSAUR5* overexpression on floral organs

**Figure 2:**
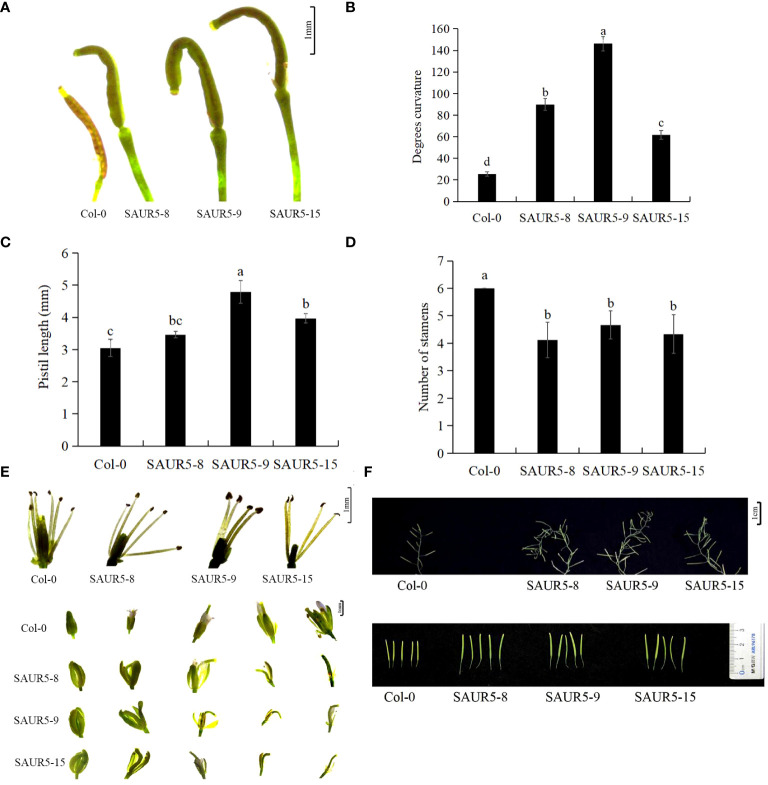
Phenotypic observation of flower organs of Col-0 and *PpSAUR5*-overexpressing *Arabidopsis* at 45 days. Each data point represents mean of the data set, and bars represent standard deviation (n = 20). Letters indicate significant differences by t test (P = 0.05). **(A)** Phenotypic observation of pistil one day after flowering. **(B)** Bending angles of pistils. **(C)** Pistil stigma length. **(D)** Number of stamens. **(E)** Phenotypic observation of stamens and buds. **(F)** Phenotypic observation of inflorescence stems and pods.

All the overexpression lines exhibited significant elongated and curved flower stigmas, with the most pronounced changes in the pistil. The stigmas in these lines were 0.75 times longer, and the bending degree was seven times higher than in Col-0, but the stigma gradually straightened at the later stage of development (**Figures 2A–C**; **Supplementary Figure S2**). Furthermore, the transgenic lines exhibited fewer stamens (four to five) than Col-0 (six). The calyx of the overexpression lines was significantly elongated and did not close tightly. In addition, the petals in these lines appeared later than Col-0, and the stigma showed a curved shape inside the bract (**Figures 2D, E**). The inflorescence stems of the transgenic plants grew spirally at the fruiting stage, and the siliques were significantly elongated with rounded tips and larger apical diameters (**Figure 2F**).

### 3.3 Effects of *PpSAUR5* overexpression on lignin content

**Figure 3:**
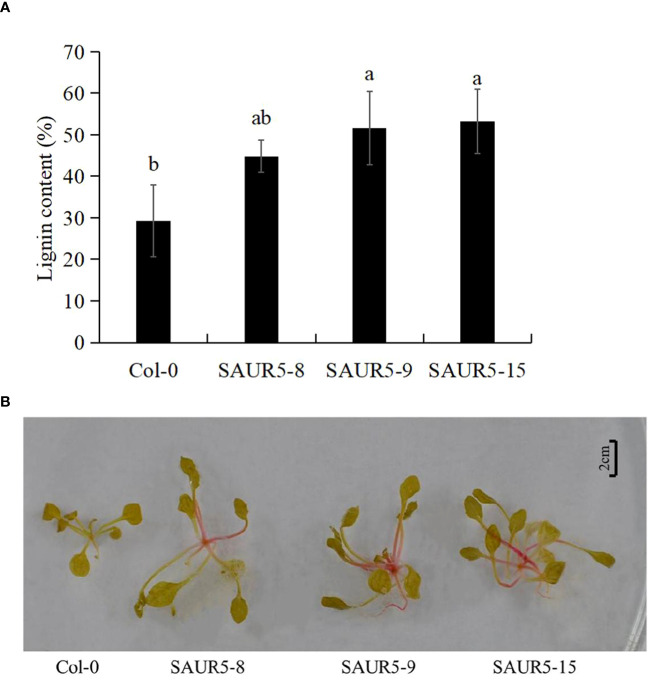
Detection of lignin content in Col-0 and *PpSAUR5*-overexpressing *Arabidopsis*. Letters indicate significant differences by t test (P = 0.05). **(A)** Lignin content in leaves of Col-0 and *PpSAUR5 Arabidopsis* overexpression lines at 20 days. Each data point represents mean of the data set and bars represent standard deviation (n = 3). **(B)** Lignin staining of Col-0 and *PpSAUR5 Arabidopsis* overexpression lines at 20 days.

Lignin is an important component of plants and is closely related to their growth. For instance, previous studies have shown a significant correlation between high lignin content and enhanced plant growth in alder (Li, 2001; Lv et al., 2018; Sun et al., 2019). In the present study, we observed that the Col-0 line had lower lignin content (29.27%) than the overexpression lines (44.80%, 51.59%, and 53.23%). Thus, *PpSAUR5* overexpression led to a significant increase in the lignin content (**Figure 3A**). Even the lignin staining was more intense (dark red) in the overexpression lines, indicating higher lignin accumulation (**Figure 3B**). These findings indicated that *PpSAUR5* might enhance overall plant growth by promoting lignin synthesis.

### 3.4 *PpSAUR5* is responsive to auxin and gibberellin

**Figure 4:**
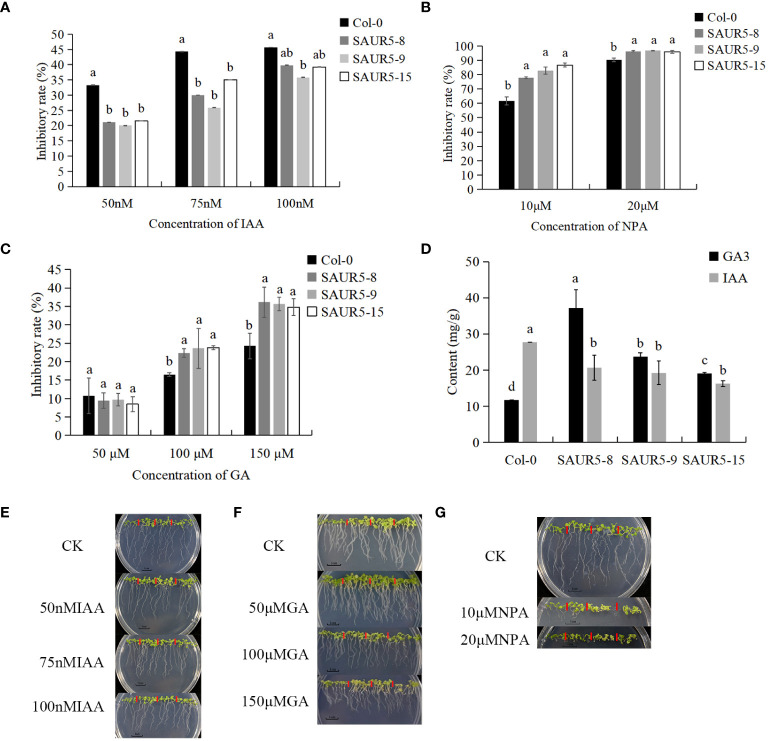
Exogenous hormone treatment and endogenous hormone changes. In **(A–C)**, the inhibition rate was root length inhibition rate. Root length inhibition rate (%) = [(control group-experimental group)/control group]. In **(E–G)**, from left to right is Col-0, *SAUR5–8*, *SAUR5–9*, and *SAUR5–15*. Letters indicate significant differences by t test (P = 0.05). **(A)** The root inhibition rate of Col-0 and *PpSAUR5 Arabidopsis*-overexpressing lines after IAA treatment for 7 days. **(B)** The root inhibition rate of Col-0 and *PpSAUR5 Arabidopsis*-overexpressing lines after NPA treatment for 7 days. **(C)** The root inhibition rate of Col-0 and *PpSAUR5 Arabidopsis*-overexpressing lines after GA treatment for 7 days. **(D)** Detection of auxin and gibberellin content in 7-day Col-0 and *PpSAUR5* overexpressing *Arabidopsis*. **(E)** Growth of Col-0 and *PpSAUR5 Arabidopsis* overexpression lines treated with IAA for 7 d. **(F)** Growth of Col-0 and *PpSAUR5 Arabidopsis* overexpression lines treated with GA for 7 days. **(G)** Growth of Col-0 and *PpSAUR5 Arabidopsis* overexpression lines treated with NPA for 7 days.

Auxin and gibberellin play an important role in promoting plant growth. Previous studies have shown that *SAUR5* family members respond to GA and IAA (Zhai et al., 2023). We assessed the response of Col-0 and *PpSAUR5* transgenic lines to varying concentrations of IAA and GA. Our results showed that, compared with the Col-0, the overexpression lines showed significantly lower and higher inhibition after IAA and NPA treatment, respectively (**Figures 4A, B**). GA at 50 μM had no significant impact on transgenic lines; however, the inhibition rate increased remarkably after treatment with 100 μM and 150 μM GA (**Figure 4C**). After 7 days of treatment, the overexpression lines exhibited significantly higher and lower levels of auxin and gibberellin, respectively, than Col-0 plants. The differences in the GA and auxin levels were the largest between Col-0 and *SAUR5–8* and Col-0 and *SAUR5–15* lines, respectively (**Figure 4D**). Thus, these findings suggested that *PpSAUR5* expression promotes gibberellin synthesis and inhibits auxin synthesis, indicating the involvement of this gene in auxin and gibberellin signal transduction.

### 3.5 Interaction between SAUR5 and PP2C.D2

PP2C.D protein is essential for plant growth and development, especially in organ growth and phototropism. It binds to SAUR proteins to negatively regulate cell expansion, thereby facilitating organ elongation (Ren et al., 2018). Thus, we hypothesized that PpSAUR5 also interacts with PP2C.D2. Y2H and luciferase complementation assays were used to validate this hypothesis.

**Figure 5:**
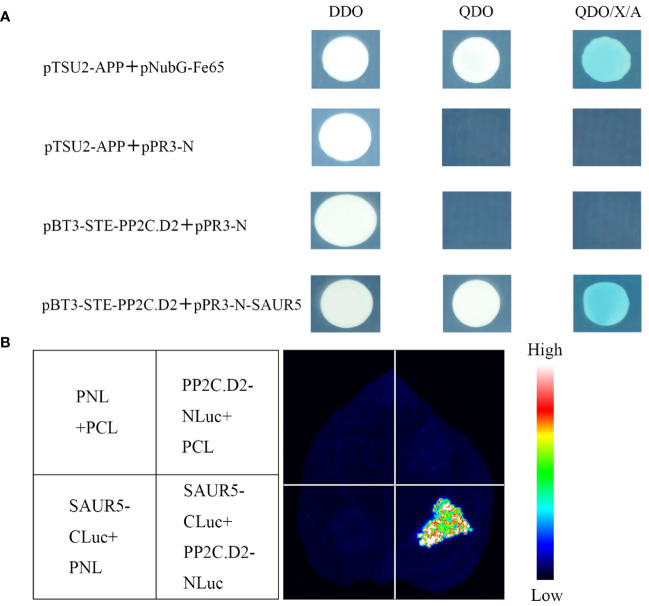
Validation of SAUR5 and PP2C.D2 interaction. **(A)** Y2H assay. DDO (SD/-Trp-Leu), QDO (SD/-Trp-Leu-Ade-His), QDO/X/A (SD/-Trp-Leu-Ade-His + X-α-gal). pTSU2-APP + pNubG-Fe65 was positive control, pTSU2-APP + pPR3-N was negative control, pBT3-STE-PP2C.D2 + pPR3-N was self-activation verification, and pBT3-STE-PP2C.D2 + pPR3-N-SAUR5 was experimental group. **(B)** Luciferase complementary assay. PP2C.D2-Nluc + SAUR5-Cluc was the experimental group. PP2C.D2-Nluc + pCL, SAUR5-Cluc + pNL, and pNL + pCL were used as the control groups.

As shown in **Figure 5A**, pBT3-STE-PP2C.D2 + pPR3-N could not grow on the QDO (SD/-Trp-Leu-Ade-His) medium, indicating that PP2C.D2 protein exhibited no autoactivation. Furthermore, pBT3-STE-PP2C.D2 + pPR3-N-SAUR5 exhibited normal growth on the QDO (SD/-Trp-Leu-Ade-His) medium and stained blue, whereas the control did not. In the luciferase complementation assay, PP2C.D2-NLuc + SAUR5-CLuc exhibited a strong fluorescence signal, whereas the control group exhibited a weak or no fluorescence signal (**Figure 5B**). These results indicated that PpSAUR5 protein interacts with PP2C.D2.

### 3.6 Analysis of transcriptome data

Next, we performed transcriptome analyses of wild-type and *SAUR5–8* lines to elucidate the role of *PpSAUR5* overexpression in inducing multiple phenotypes. Our results showed a GC content of 45%~46%, with Q20 > 99% and Q30 > 94%. The clean reads of each sample were compared with the designated reference genome for sequence alignment and obtained an alignment efficiency of >96% (**Table 1**). This finding indicated that the sequencing quality was good and ready for follow-up analyses. We identified 854 differentially expressed genes (DEGs) between the wild-type and *SAUR5–8* lines, with 753 genes upregulated and 101 genes downregulated in the overexpression line (**Supplementary Table S3**). Of these, the top 20 DEGs were selected for further analyses (**Table 2**).

**Table 1:** RNA-seq data of wild-type and *PpSAUR5*-overexpressing *Arabidopsis*.

Sample name	Mapped rate	GC(%)	Q20(%)	Q30(%)
CK-1	96.93%	46.73	99.22	94.72
CK-2	97.04%	46.18	99.21	94.64
CK-3	96.86%	45.91	99.18	94.5
SAUR5–8-1	96.57%	46.36	99.29	95.18
SAUR5–8-2	96.75%	45.91	99.36	95.69
SAUR5–8-3	96.26%	46.12	99.36	95.69

**Table 2:** Information description of common differential genes.

Gene ID	Description	KEGG pathway
AT3G30720	qua-quine starch	
AT5G39120	RmlC-like cupins superfamily protein	Selenocompound metabolism (ko00450)
AT4G25000	Alpha-amylase-like protein	Starch and sucrose metabolism (ko00500)
AT2G26010	Plant defensin 1.3	MAPK signaling pathway - plant (ko04016)
AT2G46880	Purple acid phosphatase 14	Spliceosome (ko03040)
AT5G06720	Peroxidase 2	Phenylpropanoid biosynthesis (ko00940)
AT5G06630	Hypothetical protein AXX17_AT5G06230	
AT3G29410	Terpenoid cyclases/protein prenyltransferases superfamily protein	Sesquiterpenoid and triterpenoid biosynthesis (ko00909)
AT5G35190	hypothetical protein AXX17_AT5G31360	
AT2G25160	Cytochrome P450, family 82, subfamily F, polypeptide 1	
AT3G49960	Peroxidase superfamily protein	Phenylpropanoid biosynthesis (ko00940)
AT4G13390	Proline-rich extensin-like family protein	
AT5G44430	Plant defensin 1.2C	MAPK signaling pathway - plant (ko04016)
AT4G28850	Xyloglucan endotransglucosylase/hydrolase 26	
AT1G54970	Proline-rich protein 1	
AT5G06640	Proline-rich extensin-like family protein	
AT5G39110	RmlC-like cupins superfamily protein	Selenocompound metabolism (ko00450)
AT2G32210	Cysteine-rich/transmembrane domain A-like protein	
AT5G04120	Phosphoglycerate mutase family protein	Glycolysis/gluconeogenesis (ko00010); glycine, serine and threonine metabolism (ko00260); carbon metabolism (ko01200); biosynthesis of amino acids (ko01230)
AT1G34510	Peroxidase superfamily protein	Phenylpropanoid biosynthesis (ko00940)

### 3.7 Functional analysis of DEGs

**Figure 6:**
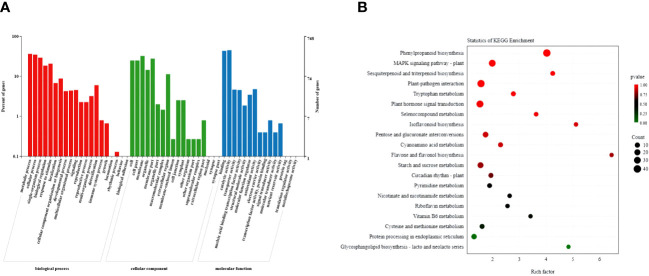
Functional analysis of DEGs in wild-type and *PpSAUR5–8*-overexpressing *Arabidopsis*. **(A)** GO functional categories of common DEGs. **(B)** The KEGG pathway of common DEGs.

The GO analysis classified the selected DEGs into three categories: biological processes, cellular components, and molecular functions (**Figure 6A**; **Supplementary Table S4**). Among the cellular components, the selected DEGs were highly enriched in cells, cell parts, cell membranes, and membrane parts. Among the biological processes, they frequently enriched in cellular processes, metabolic processes, and responses to stimuli. Among molecular functions, they frequently enriched in nucleic acid binding transcription factor activity, catalytic activity, and transporter activity. Furthermore, KEGG pathway analysis showed that the selected genes enriched in the plant hormone signal transduction, the phenylpropanoid biosynthesis, the MAPK signaling, and the plant–pathogen interaction pathways (**Figure 6B**; **Supplementary Table S5**). Based on the phenotype and hormonal response of transgenic plants, we next focused on the phenylpropanoid biosynthesis pathway, a hormone signal transduction pathway related to plant growth potential.

### 3.8 Phenylpropanoid biosynthesis pathway

**Figure 7:**
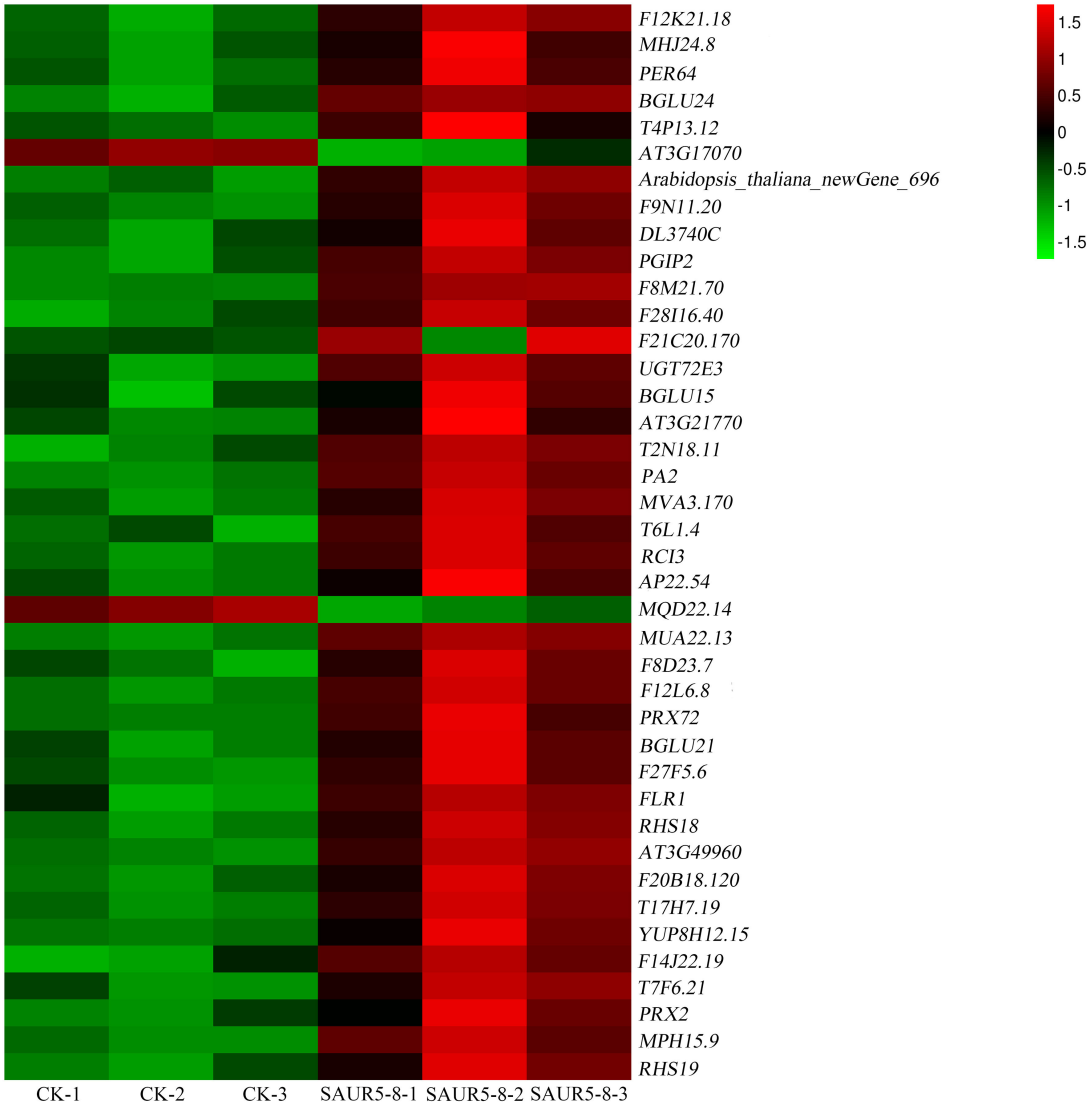
Expression of phenylalanine pathway-related genes.

Lignin is a metabolite of the phenylalanine pathway (Dong and Lin, 2021). There are three types of lignin: p-hydroxyphenyl lignin (H-lignin), guaiacyl lignin (G-lignin), and syringyl lignin (S-lignin). The catalase enzyme catalyzes the process of lignin synthesis (Wei and Song, 2001). Among the 854 DEGs identified earlier, 40 DEGs were related to phenylalanine metabolism. Of these, 38 DEGs were upregulated in the transgenic line, including *PER64* (AT5G42180), *F14J22.19* (AT1G49570), *F8D23.7* (AT2G18150), *T4P13.12* (AT3G01190), *F12K21.18* (AT1G34510), *YUP8H12.15* (AT1G05240), *F8M21.70* (AT5G15180), *T6L1.4* (AT1G68850), *MVA3.170* (AT5G17820), *PRX72* (AT5G66390), *RCI3* (AT1G05260), *T7F6.21* (AT2G39040), *T2N18.11* (AT2G37130), *T17H7.19* (AT1G30870), AT3G49960, *PRX2* (AT1G05250), *F28I16.40* (AT5G19890), *MUA22.13* (AT5G14130), AT3G21770, *F20B18.120* (AT4G26010), *AP22.54* (AT4G36430), *MPH15.9* (AT5G06730), *F27F5.6* (AT1G44970), *MHJ24.8* (AT5G64100), *F21C20.170* (AT4G20820), *RHS18* (AT5G22410), *RHS19* (AT5G67400), *DL3740C* (AT4G15390), *F12L6.8* (AT2G39420), *PA2* (AT5G06720), *F9N11.20* (AT4G30170), *Arabidopsis thaliana newGene 696, UGT72E3* (AT5G26310), *BGLU15* (AT2G44450), *BGLU24* (AT5G28510), *PGIP2* (AT5G06870), *BGLU21* (AT1G66270), and *FLR1* (AT3G12145). *MQD22.14* (AT5G47000) and AT3G17070 were downregulated in the transgenic line (**Figure 7**; **Supplementary Table S6**). This finding indicated that *PpSAUR5* overexpression might mediate lignin synthesis by modulating the phenylpropanoid metabolic pathway.

### 3.9 Plant hormone signal transduction pathway

**Figure 8:**
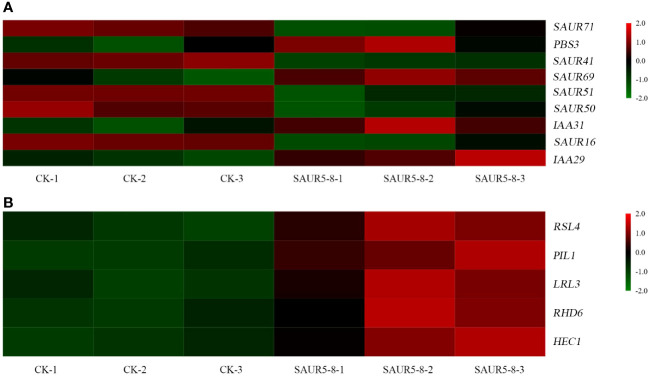
Expression of auxin **(A)** and gibberellin **(B)** related genes.

In the plant hormone signal transduction pathway, the DEGs were primarily enriched in auxin signal transduction, gibberellin signal transduction, zeatin synthesis, and diterpenoid biosynthesis pathways (**Figure 8**; **Supplementary Table S7**). Five *SAUR* family genes, *SAUR16* (AT4G38860), *SAUR41* (AT1G16510), *SAUR71* (AT1G56150), *SAUR51* (AT1G75580), and *SAUR50* (AT4G34760), were downregulated in the auxin signal transduction pathway, whereas *SAUR69* (AT5G10990) was upregulated. Moreover, two IAA genes (*IAA31* and *IAA29*), one Gretchen Hagen 3 (*GH3*) gene (*PBS3*), and five genes in the gibberellin signal transduction pathway (*RSL4*, *PIL1*, *LRL3*, *RHD6*, and *HEC1*) were also upregulated.

### 3.10 qRT-PCR validation of DEGs

**Figure 9:**
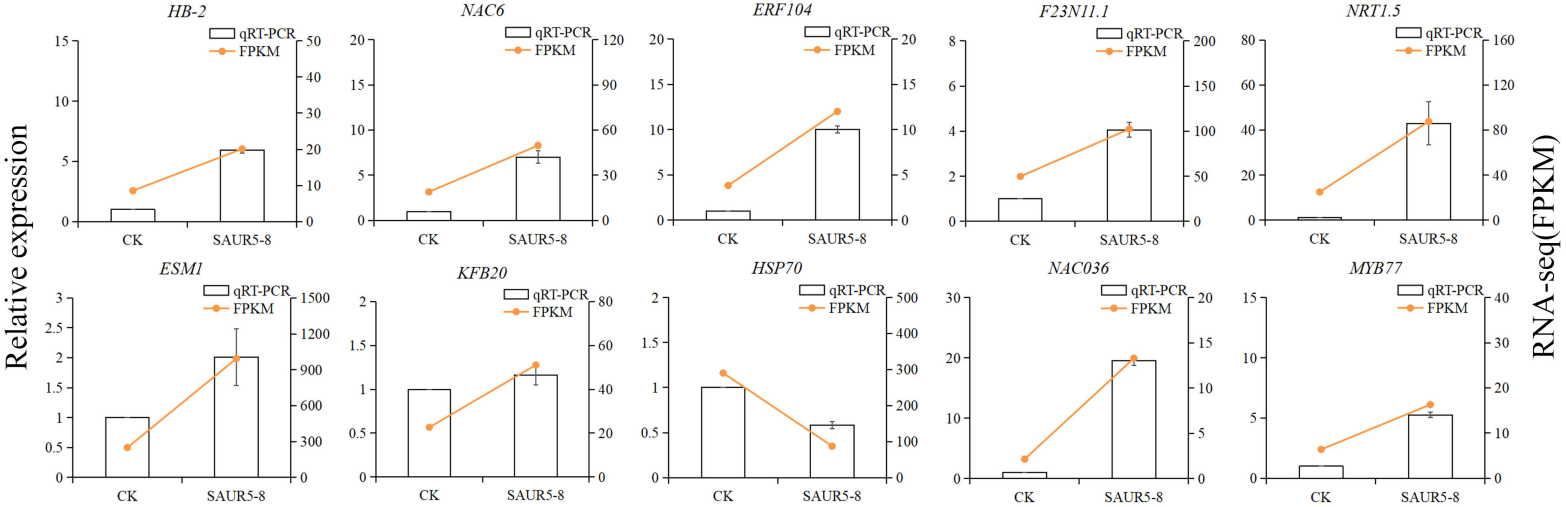
qRT-PCR results of DEGs.

There were 10 DEGs randomly selected for qRT-PCR validation. The internal reference gene was *ubq5*. The fragments per kilobase of transcript per million mapped read (FPKM) values measured in the transcriptome were consistent with the relative expression pattern of these 10 genes (**Figure 9**), indicating that RNA-seq data have good reliability.

## 4 Discussion

Auxin is an essential phytohormone that regulates plant growth both directly and indirectly. The gibberellin signal transduction pathway comprises the DELLA protein, which inhibits the gibberellin response by binding to the sequestering transcription factor, similar to the auxin signaling pathway (Stamm and Kumar, 2013). The expression of GA biosynthetic genes is regulated by Aux/IAA and ADP ribosylation factor (Frigerio et al., 2006). Auxin regulates gibberellin-induced root growth by degrading the DELLA protein (Fu and Harberd, 2003). On the contrary, GA regulates the PIN-FORMED (PIN) protein, impacting the distribution of auxin in the root tip (Willige et al., 2011). In tomato, *SlARF7* has been found to induce auxin and gibberellin responses during fruit development (de Jong et al., 2011).

The expression of the *SAUR* gene family members can be rapidly modulated by auxin treatment. It is one of the auxin response gene families. Eighty members of the *SAUR* gene family, including two pseudogenes, have been identified in *Arabidopsis*. However, only a few *SAUR* genes have been functionally characterized. A previous study reported *AAM1*, a member of the SAUR family, to be closely associated with apical hook development in *Arabidopsis* (Park et al., 2007). *AtSAUR63* overexpression has been found to elevate auxin levels in *Arabidopsis* hypocotyls and promote hypocotyl and stamen filament elongation (Chae et al., 2012). *SAUR41* overexpression has been found to result in hypocotyl elongation, distorted inflorescence stems, enlarged petals, and an increase in the number of lateral roots in *Arabidopsis* (Kong et al., 2013). Early leaf decay has been reported in plants overexpressing *SAUR71* and *SAUR72* (Zhou et al., 2018; Jia, 2020). Research on the *SAUR* family has mainly focused on soybean, rice, tomato, potato, maize, and other plants. However, studies on the SAUR family members of peach have been rarely reported (Hagen and Guilfoyle, 2002; Ma et al., 2017; Guo et al., 2019).

Several studies have shown that the biological clock regulates auxin signal transduction and the expressions of many auxin-induced genes, resulting in varying plant sensitivity to auxin at different times of the day. The asymmetry in auxin accumulation is related to stem bending growth (Covington and Harmer, 2007; Chae et al., 2012). However, whether the distortion of the inflorescence stem observed in the overexpression lines in the current study is related to the circadian clock signal or the optical signal still remains to be investigated.

Paclobutrazol effectively inhibits the growth of new shoots and is used to control the growth of peach trees. Spraying paclobutrazol on peach trees reduces gibberellin activity and inhibits the expression of genes involved in gibberellin synthesis (Zhou, 2020). The non-synonymous single-nucleotide mutation in the gibberellin receptor gibberellin insensitive dwarf 1 (GID1) of the dwarf variety Shouxing peach inhibited its interaction with DELLA, thus making this plant insensitive to GA (Cheng et al., 2019). Overexpression of *SAUR76–78* reportedly results in low sensitivity to ethylene treatment, suggesting the possible involvement of these genes in ethylene signaling (Li et al., 2015). The *SAUR* family might act as a node in the auxin and gibberellin signaling pathways. *MeSAUR1* is highly expressed in cassava roots. Its expression increases after induction by IAA, gibberellin, and ethylene (Ma et al., 2017). *AtSAUR36* is induced by auxin but inhibited by gibberellin. Its expression can also be affected by polyphenols and exogenous ABA treatment (Stamm and Kumar, 2013). *PIL1* (AT2G46970) interacts with *COP1* to regulate the circadian rhythm, photomorphogenesis, and hypocotyl elongation (Luo et al., 2014). In the current study, the results of the transcriptome analysis showed that *PpSAUR5* upregulated *PIL1*, which is involved in the gibberellin synthesis pathway (**Figure 8B**). In addition, *PpSAUR5* was found to be responsive to auxin and gibberellin, as evident by increased gibberellin levels and decreased auxin levels in the overexpression plants (**Figure 4**). These results indicated that *PpSAUR5* is involved in auxin and gibberellin signal transduction pathways.

Jiang (2022) found significant pistil elongation in the *PpIDD11* overexpression lines. Another study reported that *AtSAUR63* promotes stamen filament elongation (Chae et al., 2012). However, to the best of our knowledge, there are no reports on the impact of *SAUR* family members on the number of stamens. In the current study, we found that *PpSAUR5* overexpression led to a decrease in the number of stamens and elongation and bending of pistil stigmas (**Figure 2**). We also observed a significant increase in the diameter and leaf area of rosette leaf discs in the overexpression lines (**Figures 1E, F**). In addition, *PpSAUR5* promoted the expansion of floral organs and the distortion of inflorescence stems during flowering (**Figure 2**), which was consistent with the phenotype of the plants overexpressing *SAUR41* (Kong et al., 2013). The *PP2C.D* (D-type protein phosphatase 2C) family is essential for plant growth and development processes, such as organ growth. *SAUR* has previously been found to activate PM H^+^-ATPase and promote cell expansion by inhibiting PP2C.D (D-type protein phosphatase 2C) activity (Spartz et al., 2017). In a previous study, overexpression of *GFP-SAUR19* was found to inhibit the growth defects induced by *PP2C.D5* (Ren et al., 2018). In line with these studies, in the present study, we found that SAUR5 interacted with PP2C.D2 (**Figure 5**), indicating that *PpSAUR5* might impact shoot growth in peach by modulating PP2C.D2 activity.

We identified a total of 854 DEGs from the transcriptome data of overexpression and wild-type lines. These DEGs were found to play regulatory roles in chlorophyll synthesis, auxin and gibberellin signaling, and lignin synthesis. There were 40 DEGs were found to be associated with the phenylalanine metabolic pathway (**Figure 7**; **Supplementary Table S6**). In addition, we found higher lignin content and lower chlorophyll content in the overexpression lines than in the Col-0 lines, suggesting the potential involvement of *PpSAUR5* in lignification and chlorophyll accumulation.

Taken together, *PpSAUR5* might promote plant growth by modulating the auxin and gibberellin signal transduction pathways. However, the process of plant growth and development is complex, and the specific regulatory mechanisms underlying *PpSAUR5* action still need to be investigated.

## Data availability statement

The original contributions presented in the study are included in the article/**Supplementary Files**, further inquiries can be directed to the corresponding authors.

## Author contributions

X-ML: Writing – original draft. H-HZ: Writing – original draft. X-HA: Writing – review & editing. HZ: Writing – review & editing. XZ: Writing – review & editing. PW: Writing – review & editing. HC: Writing – review & editing. YT: Writing – review & editing.

## Supplementary material

Supplementary Figure 1 Rosette phenotypes of Col-0 and *PpSAUR5*-overexpressing *Arabidopsis* lines grown in soil for 20 days.

Supplementary Figure 2 Silique developmental stages in Col-0 and *PpSAUR5*-overexpressing lines. The siliques at 1, 3, 6, 9 and 12 days after flowering are displayed.

## References

[B1] Achard P. Genschik P. (2009). Releasing the brakes of plant growth: how GAs shutdown DELLA proteins. J. Exp. Bot. 60, 1085–1092. doi: 10.1093/jxb/ern301 19043067

[B2] Béziat C. Kleine-Vehn J. (2018). The road to auxin-dependent growth repression and promotion in apical hooks. Curr. Biol. 28, R519–R525. doi: 10.1016/j.cub.2018.01.069 29689235

[B3] Chae K. Isaacs C. G. Reeves P. H. Maloney G. S. Muday G. K. Nagpal P.. (2012). *Arabidopsis* SMALL AUXIN UP RNA63 promotes hypocotyl and stamen filament elongation. Plant J. 71, 684–697. doi: 10.1111/j.1365-313X.2012.05024.x 22507274

[B4] Cheng J. Zhang M. Tan B. Jiang Y. Zheng X. Ye X.. (2019). A single nucleotide mutation in GID1c disrupts its interaction with DELLA1 and causes a GA-insensitive dwarf phenotype in peach. Plant Biotechnol. J. 17, 1723–1735. doi: 10.1111/pbi.13094 30776191 PMC6686139

[B5] Clough S. J. Bent A. F. (1998). Floral dip: a simplified method for Agrobacterium-mediated transformation of *Arabidopsis thaliana* . Plant J. 16, 735–743. doi: 10.1046/j.1365-313x.1998.00343.x 10069079

[B6] Covington M. F. Harmer S. L. (2007). The circadian clock regulates auxin signaling and responses in *Arabidopsis* . PLoS Biol. 5, e222. doi: 10.1371/journal.pbio.0050222 17683202 PMC1939880

[B7] de Jong M. Wolters-Arts M. García-Martínez J. L. Mariani C. Vriezen W. H. (2011). The Solanum lycopersicum AUXIN RESPONSE FACTOR 7 (*SlARF7*) mediates cross-talk between auxin and gibberellin signalling during tomato fruit set and development. J. Exp. Bot. 62, 617–626. doi: 10.1093/jxb/erq293 20937732 PMC3003806

[B8] Dong N. Q. Lin H. X. (2021). Contribution of phenylpropanoid metabolism to plant development and plant-environment interactions. J. Integr. Plant Biol. 63, 180–209. doi: 10.1111/jipb.13054 33325112

[B9] Dorcey E. Urbez C. Blázquez M. A. Carbonell J. Perez-Amador M. A. (2009). Fertilization-dependent auxin response in ovules triggers fruit development through the modulation of gibberellin metabolism in *Arabidopsis* . Plant J. 58, 318–332. doi: 10.1111/j.1365-313X.2008.03781.x 19207215

[B10] Frigerio M. Alabadií D. Peírez-Goímez J. Garciía-Caírcel L. Phillips A. L. Hedden P.. (2006). Transcriptional regulation of gibberellin metabolism genes by auxin signaling in *arabidopsis* . Plant Physiol. 142, 553–563. doi: 10.1104/pp.106.084871 16905669 PMC1586059

[B11] Fu X. Harberd N. P. (2003). Auxin promotes *Arabidopsis* root growth by modulating gibberellin response. Nature 421, 740–743. doi: 10.1038/nature01387 12610625

[B12] Gil P. Liu Y. Orbović V. Verkamp E. Poff K. L. Green P. J. (1994). Characterization of the auxin-inducible *SAUR-AC1* gene for use as a molecular genetic tool in *Arabidopsis* . Plant Physiol. 104, 777–784. doi: 10.1104/pp.104.2.777 8159792 PMC159258

[B13] Gray W. M. Ostin A. Sandberg G. Romano C. P. Estelle M. (1998). High temperature promotes auxin-mediated hypocotyl elongation in *Arabidopsis* . Proc. Natl. Acad. Sci. U.S.A. 95, 7197–7202. doi: 10.1073/pnas.95.12.7197 9618562 PMC22781

[B14] Guo D. Du M. Zhou B. Liu Y. Zhao M. (2019). Identification and bioinformatics analysis of the maize SAUR gene family. J. Plant Genet. Resour. 20, 90–99. doi: 10.13430/j.cnki.jpgr.20180707001

[B15] Hagen G. Guilfoyle T. (2002). Auxin-responsive gene expression: genes, promoters and regulatory factors. Plant Mol. Biol. 49, 373–385. doi: 10.1023/A:1015207114117 12036261

[B16] Hou K. Wu W. Gan S.-S. (2013). *SAUR36*, a SMALL AUXIN UP RNA gene, is involved in the promotion of leaf senescence in *arabidopsis* . Plant Physiol. 161, 1002–1009. doi: 10.1104/pp.112.212787 23250625 PMC3560998

[B17] Hu J. Israeli A. Ori N. Sun T.-P. (2018). The interaction between DELLA and ARF/IAA mediates crosstalk between gibberellin and auxin signaling to control fruit initiation in tomato. Plant Cell 30, 1710–1728. doi: 10.1105/tpc.18.00363 30008445 PMC6139683

[B18] Jia S. (2020). Cloning and preliminary functional characterisation of *SAUR71* gene in creeping bentgrass. Beijing: Beijing Forestry University.

[B19] Jiang Y. (2022). *PpIDD4*, *PpIDD12* and *PpIDD13* are involved in DELLA-dependent feedback regulation of gibberellin synthesis and *PpIDD11* regulates pistil stigma elongation in peach. Zhengzhou, Henan: Henan Agricultural University.

[B20] Kant S. Bi Y. M. Zhu T. Rothstein S. J. (2009). *SAUR39*, a small auxin-up RNA gene, acts as a negative regulator of auxin synthesis and transport in rice. Plant Physiol. 151, 691–701. doi: 10.1104/pp.109.143875 19700562 PMC2754634

[B21] Kathare P. K. Dharmasiri S. Dharmasiri N. (2018). *SAUR53* regulates organ elongation and apical hook development in *Arabidopsis* . Plant Signal Behav. 13, e1514896. doi: 10.1080/15592324.2018.1514896 30260266 PMC6204813

[B22] Knauss S. Rohrmeier T. Lehle L. (2003). The auxin-induced maize gene *zmSAUR2* encodes a short-lived nuclear protein expressed in elongating tissues*. J. Biol. Chem. 278, 23936–23943. doi: 10.1074/jbc.M212585200 12695517

[B23] Kong Y. Zhu Y. Gao C. She W. Lin W. Chen Y.. (2013). Tissue-specific expression of SMALL AUXIN UP RNA41 differentially regulates cell expansion and root meristem patterning in *Arabidopsis* . Plant Cell Physiol. 54, 609–621. doi: 10.1093/pcp/pct028 23396598

[B24] Lee R. D. Cho H. T. (2013). Auxin, the organizer of the hormonal/environmental signals for root hair growth. Front. Plant Sci. 4, 448. doi: 10.3389/fpls.2013.00448 24273547 PMC3824147

[B25] Li W. (2001). Study on the relationship between PAL, lignin and growth of different clones of alder at seedling stage. Beijing: Chinese Academy of Forestry.

[B26] Li Z. G. Chen H. W. Li Q. T. Tao J. J. Bian X. H. Ma B.. (2015). Three SAUR proteins SAUR76, SAUR77 and SAUR78 promote plant growth in *Arabidopsis* . Sci. Rep. 5, 12477. doi: 10.1038/srep12477 26207341 PMC4513569

[B27] Li S. Peng F. Xiao Y. Gong Q. Bao Z. Li Y.. (2020). Mechanisms of high concentration valine-mediated inhibition of peach tree shoot growth. Front. Plant Sci. 11, 603067. doi: 10.3389/fpls.2020.603067 33193558 PMC7658097

[B28] Luo Q. Lian H.-L. He S.-B. Li L. Jia K.-P. Yang H.-Q. (2014). COP1 and phyB physically interact with PIL1 to regulate its stability and photomorphogenic development in *arabidopsis* . Plant Cell 26, 2441–2456. doi: 10.1105/tpc.113.121657 24951480 PMC4114944

[B29] Lv T. Shen R. Yang H. Fan W. Zhang R. Wang L.. (2018). Effects of lignin under applied organic fertiliser on root vigour and inter-root soil microbiology of Pingyi sweet tea. J. Shandong Agric. Univ. (Natural Sci. Edition) 49, 561–565. doi: 10.3969/j.issn.1000-2324.2018.04.003

[B30] Ma P. Chen X. Liu C. Meng Y. Xia Z. Zeng C.. (2017). *MeSAUR1*, encoded by a small auxin-up RNA gene, acts as a transcription regulator to positively regulate ADP-glucose pyrophosphorylase small subunit1a gene in cassava. Front. Plant Sci. 8, 1315. doi: 10.3389/fpls.2017.01315 28824663 PMC5534448

[B31] McClure B. A. Guilfoyle T. (1987). Characterization of a class of small auxin-inducible soybean polyadenylated RNAs. Plant Mol. Biol. 9, 611–623. doi: 10.1007/BF00020537 24277197

[B32] Newman T. C. Ohme-Takagi M. Taylor C. B. Green P. J. (1993). DST sequences, highly conserved among plant *SAUR* genes, target reporter transcripts for rapid decay in tobacco. Plant Cell 5, 701–714. doi: 10.1105/tpc.5.6.701 8329900 PMC160307

[B33] Park J.-E. Kim Y.-S. Yoon H.-K. Park C.-M. (2007). Functional characterization of a small auxin-up RNA gene in apical hook development in *Arabidopsis* . Plant Sci. 172, 150–157. doi: 10.1016/j.plantsci.2006.08.005

[B34] Ren H. Park M. Y. Spartz A. K. Wong J. H. Gray W. M. (2018). A subset of plasma membrane-localized PP2C.D phosphatases negatively regulate SAUR-mediated cell expansion in *Arabidopsis* . PLoS Genet. 14, e1007455. doi: 10.1371/journal.pgen.1007455 29897949 PMC6016943

[B35] Ross J. J. O’Neill D. P. Smith J. J. Kerckhoffs L. H. Elliott R. C. (2000). Evidence that auxin promotes gibberellin A1 biosynthesis in pea. Plant J. 21, 547–552. doi: 10.1046/j.1365-313x.2000.00702.x 10758505

[B36] Sandalio L. M. Rodríguez-Serrano M. Romero-Puertas M. C. (2016). Leaf epinasty and auxin: A biochemical and molecular overview. Plant Sci. 253, 187–193. doi: 10.1016/j.plantsci.2016.10.002 27968987

[B37] Spartz A. K. Lor V. S. Ren H. Olszewski N. E. Miller N. D. Wu G.. (2017). Constitutive expression of *arabidopsis* SMALL AUXIN UP RNA19 (*SAUR19*) in tomato confers auxin-independent hypocotyl elongation. Plant Physiol. 173, 1453–1462. doi: 10.1104/pp.16.01514 27999086 PMC5291034

[B38] Stamm P. Kumar P. P. (2013). Auxin and gibberellin responsive *Arabidopsis* SMALL AUXIN UP RNA36 regulates hypocotyl elongation in the light. Plant Cell Rep. 32, 759–769. doi: 10.1007/s00299-013-1406-5 23503980

[B39] Stepanova A. N. Robertson-Hoyt J. Yun J. Benavente L. M. Xie D. Y. Dolezal K.. (2008). TAA1-mediated auxin biosynthesis is essential for hormone crosstalk and plant development. Cell 133, 177–191. doi: 10.1016/j.cell.2008.01.047 18394997

[B40] Stortenbeker N. Bemer M. (2019). The *SAUR* gene family: the plant’s toolbox for adaptation of growth and development. J. Exp. Bot. 70, 17–27. doi: 10.1093/jxb/ery332 30239806

[B41] Sun R. Zhai M. Li H. Li M. Wang F. Zhang H. (2019). Study on the relationship between lignin synthesis and growth and development of Pokeweed. Modern Agric. Sci. Technol. 5, 126–127. doi: 10.3969/j.issn.1007-5739.2019.05.071

[B42] Tao Y. Ferrer J. L. Ljung K. Pojer F. Hong F. Long J. A.. (2008). Rapid synthesis of auxin via a new tryptophan-dependent pathway is required for shade avoidance in plants. Cell 133, 164–176. doi: 10.1016/j.cell.2008.01.049 18394996 PMC2442466

[B43] Van Acker R. Vanholme R. Storme V. Mortimer J. C. Dupree P. Boerjan W. (2013). Lignin biosynthesis perturbations affect secondary cell wall composition and saccharification yield in *Arabidopsis thaliana* . Biotechnol. Biofuels 6, 46. doi: 10.1186/1754-6834-6-46 23622268 PMC3661393

[B44] van Mourik H. van Dijk A. D. J. Stortenbeker N. Angenent G. C. Bemer M. (2017). Divergent regulation of *Arabidopsis SAUR* genes: a focus on the *SAUR10*-clade. BMC Plant Biol. 17, 245. doi: 10.1186/s12870-017-1210-4 29258424 PMC5735953

[B45] Wei J. H. Song Y. R. (2001). Recent advances in study of lignin biosynthesis and manipulation. Acta Botanica Sin. 43, 771–779.

[B46] Willige B. C. Isono E. Richter R. Zourelidou M. Schwechheimer C. (2011). Gibberellin regulates PIN-FORMED abundance and is required for auxin transport–dependent growth and development in *arabidopsis thaliana* . Plant Cell 23, 2184–2195. doi: 10.1105/tpc.111.086355 21642547 PMC3160035

[B47] Wong J. H. Spartz A. K. Park M. Y. Du M. Gray W. M. (2019). Mutation of a conserved motif of *PP2C.D* phosphatases confers *SAUR* immunity and constitutive activity. Plant Physiol. 181, 353–366. doi: 10.1104/pp.19.00496 31311832 PMC6716246

[B48] Xia L. Mar Marquès-Bueno M. Bruce C. G. Karnik R. (2019). Unusual roles of secretory SNARE SYP132 in plasma membrane H(+)-ATPase traffic and vegetative plant growth. Plant Physiol. 180, 837–858. doi: 10.1104/pp.19.00266 30926657 PMC6548232

[B49] Xie R. Dong C. Ma Y. Deng L. He S. Yi S.. (2015). Comprehensive analysis of *SAUR* gene family in citrus and its transcriptional correlation with fruitlet drop from abscission zone A. Funct. Integr. Genomics 15, 729–740. doi: 10.1007/s10142-015-0450-3 26115718

[B50] Xu Y. X. Xiao M. Z. Liu Y. Fu J. L. He Y. Jiang D. A. (2017). The small auxin-up RNA *OsSAUR45* affects auxin synthesis and transport in rice. Plant Mol. Biol. 94, 97–107. doi: 10.1007/s11103-017-0595-7 28321650

[B51] Zhai H. H. Zhai Y. Tian Y. Zhang Y. Yang L. Wen Z.. (2023). Peach SAUR family gene analysis and functional identification of *PpSAUR5* . Hortic. J. 50, 1–14. doi: 10.16420/j.issn.0513-353x.2021-0932

[B52] Zhou X. (2020). Study on the effect and related molecular mechanism of paclobutrazol on inhibiting the growth of peach shoots. Baoding, Hebei: Hebei Agricultural University.

[B53] Zhou J. Wen Z. Mei Y. Y. Wang N. N. (2018). Mechanism of SAUR72 in the regulation of leaf senescence in *Arabidopsis thaliana* . J. Plant Physiol. 54, 379–385. doi: 10.13592/j.cnki.ppj.2018.1001

