# Supplementary figures and images for: PpSAUR5 promotes plant growth by regulating lignin and hormone pathways
Source: Front Plant Sci. 2024 Jun 25;15:1291693. doi: 10.3389/fpls.2024.1291693 (PMC11231374; doi:10.3389/fpls.2024.1291693)

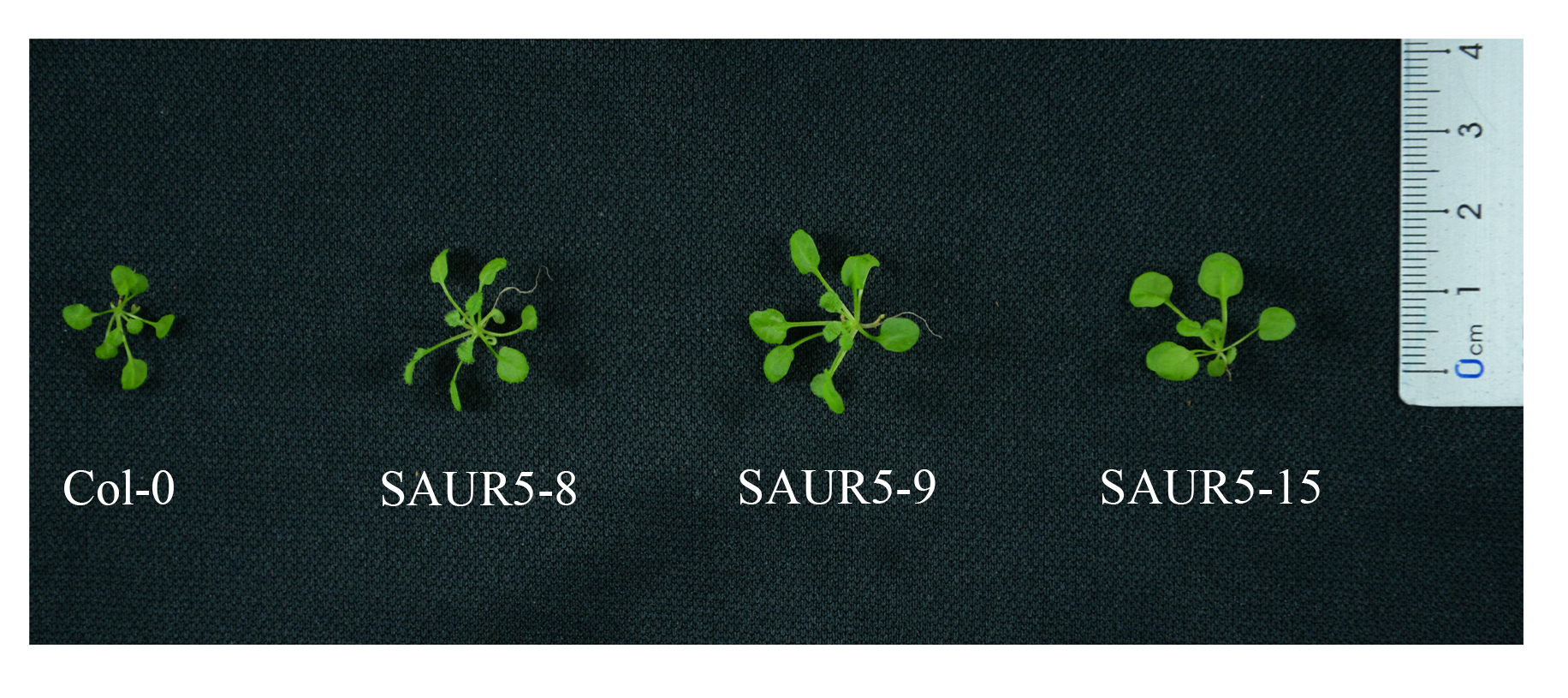

Supplement: Supplementary Figure 1 — Rosette phenotypes of Col-0 and PpSAUR5-overexpressing Arabidopsis lines grown in soil for 20 days. [file Image_1.jpeg]

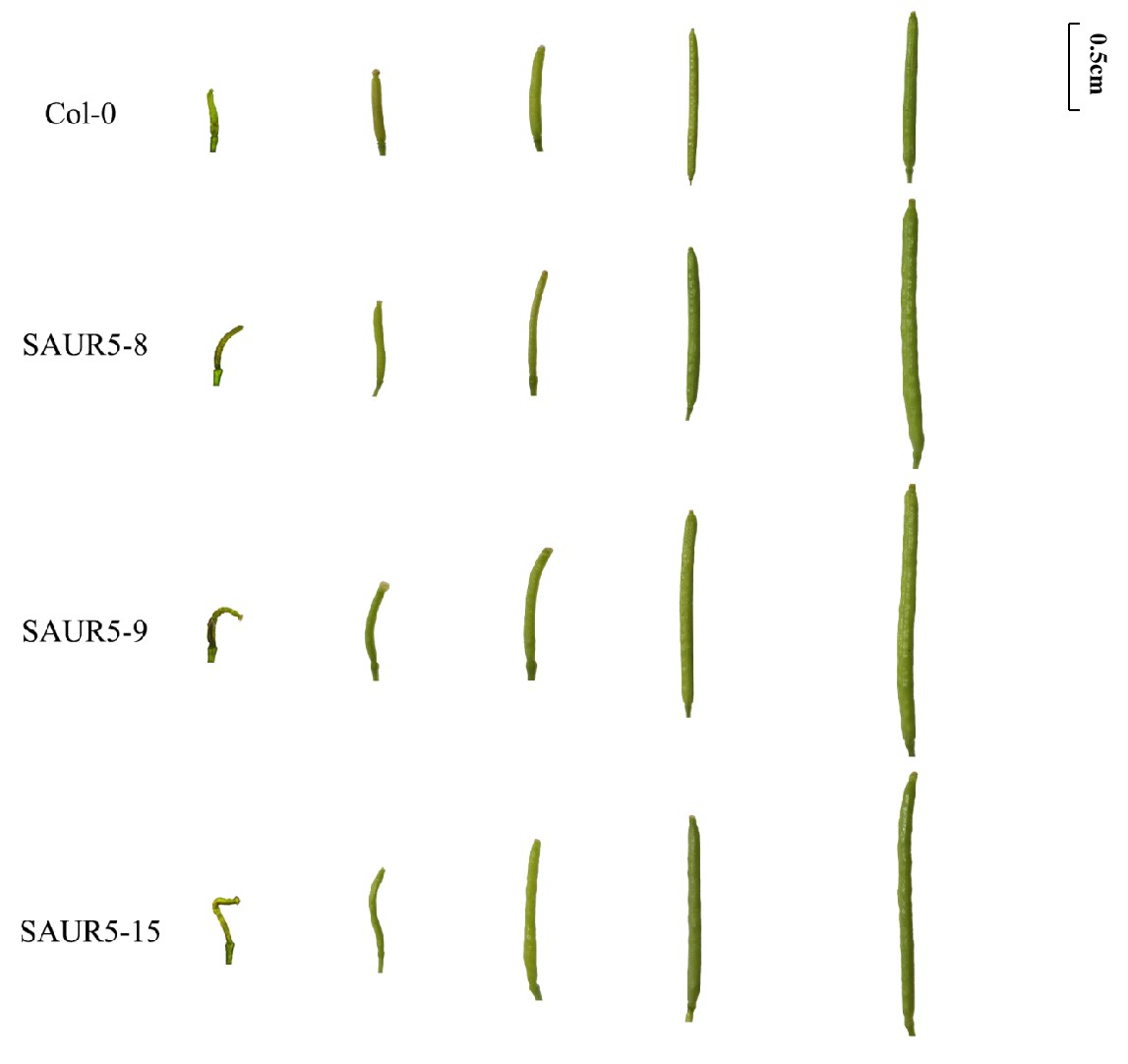

Supplement: Supplementary Figure 2 — Silique developmental stages in Col-0 and PpSAUR5-overexpressing lines. The siliques at 1, 3, 6, 9 and 12 days after flowering are displayed. [file Image_2.jpeg]
