# Supplementary material for: PpSAUR5 promotes plant growth by regulating lignin and hormone pathways
Source: Front Plant Sci. 2024 Jun 25;15:1291693. doi: 10.3389/fpls.2024.1291693 (PMC11231374; doi:10.3389/fpls.2024.1291693)
Supplement: Supplementary file 3 [file Table_1.docx]

**Supplementary Table S1. Primers for constructing expression vectors.**

| Name | Primer sequence (5'→3') |
| --- | --- |
| pRI-*PpSAUR5*-F | GGATCCATGTCAGCCGGACTGGGAAAAT |
| pRI-*PpSAUR5*-R | GAATTCTTACCAAATTGTTTTATCTGAAAATCCAC |
| CaMV35s-F | GCTCCTACAAATGCCATCA |
| *PpSAUR5*-R | TTACCAAATTGTTTTATCTGAAAAT |
