# Supplementary material for: PpSAUR5 promotes plant growth by regulating lignin and hormone pathways
Source: Front Plant Sci. 2024 Jun 25;15:1291693. doi: 10.3389/fpls.2024.1291693 (PMC11231374; doi:10.3389/fpls.2024.1291693)
Supplement: Supplementary file 4 [file Table_2.docx]

**Supplementary Table S2. The primers used for qRT-PCR**

| ID | Gene name | Forward primer | Reverse primer |
| --- | --- | --- | --- |
| Prupe.1G368100 | PpSAUR5 | GCTCCTTGTAGAAGCCGAGG | CGGCCCAGAAATCGAGGTTA |
| AT3G62250 | ubq5 | AACCCTTGAGGTTGAATCATCC | GTCCTTCTTTCTGGTAAACGT |
| AT3G12580 | HSP70 | TCACTCCTCTGTCCTTGGGT | TCCTTTGTTCGTGCCCTCTC |
| AT1G80440 | KFB20 | AGCTTCCTCCGATTCCTGGTCAAA | CACGCGCCATTTGGAAGTGAGAAA |
| AT4G16780 | HB-2 | CAAACGGAGGTAGACTGCGA | GTCAAAGTAGTGGGTGGGCT |
| AT3G50060 | MYB77 | TTTGCTCCGGTGGATACTGG | CTGCGGAACCACCGTAAAAC |
| AT5G61600 | ERF104 | GCGGCTAGGGTTAAAGTGGA | CCATCTCCTGCTCCCACATC |
| AT5G39610 | NAC6 | TGTCCACGAGTCCAAAGACG | TGTACCGGACGAATCACGAC |
| AT2G17040 | NAC036 | TGGAGCAAAGGGCAGAGATG | TACTTGAGGAGCACGGAACG |
| AT3G14210 | ESM1 | AACGCCGTTCTTCGTAGGAT | TTCGAGTGCACGTTTCCCTT |
| AT1G32450 | NRT1.5 | AGGATCACATGCCTGGTTGG | ACGCAGCCAGGAGGAAATAG |
| AT2G20670 | F23N11.1 | AAAAGCGAGATGCGAGTGGT | CCGACGTAAACCTCAGGCAA |
